
JOURNAL INFORMATION
==============================
NLM Title Abbreviation: Atlanta Med Surg J (1884)
NLM Journal ID: 101251623

Atlanta Medical and Surgical Journal (1884)

Publisher: Biomedical Journal Digitization Project

ARTICLE INFORMATION
==============================
PMCID: PMC9199296
PMID: 35829593
Article version: 1
Subjects: Editorial

Christian Science and Death

Print publication date: 1898 Dec
Volume: 15
Volume Series: New Ser
Issue: 10
First page: 681
Last page: 685
License: This work is free of known copyright restrictions. For more information, please see PMC Back Issue Digitization.
License URL: https://creativecommons.org/publicdomain/mark/1.0/

==============================

### *EDITORIAL NOTES AND COMMENTS.*

---

The Business office of THE JOURNAL is 305, 306 Fitten Building.

The Editorial office is Room 401, 402 Grand Opera House.

Address all Business communications to Dr. M. B. Hutchins, Mgr.

Make remittances payable to THE ATLANTA MEDICAL AND SURGICAL JOURNAL.

On matters pertaining to the Editorial and Original communications address Dr. Dunbar Roy, Grand Opera House, Atlanta.

Reprints of original articles will be furnished at cost price. Requests for the same should always be made *on the manuscript*.

We will present, post-paid, on request, to each contributor of an original article, twenty (20) marked copies of THE JOURNAL containing such article.

---

### CHRISTIAN SCIENCE AND DEATH.

UNDER this editorial caption the *Boston Medical and Surgical Journal* has expressed itself in no uncertain terms, and the views thus held correspond entirely with our own. The era of "fads" will never cease, and none seem so mythically winsome as the much heralded Christian Science. What Boston is to the North as a center for this peculiar sect of people, Atlanta is to the South in a similar relation. Strange deluded people to be led away by such strange nonsensical fanaticism! But "religious craze" is the most common of all insanities, and we judge that there will be insane people as long as the world shall last. Those who die under the administration of the Christian Science faith believe that such a death is that of a martyr and the survivors go on rejoicing that one more has been added to a righteous band. Only in the last month has the death of a victim been chronicled in the daily newspaper right here in Atlanta who refused to change a "spiritual administrator" for a physician in good standing. The neighbors raised a huge cry against the practice of such barbarity, but his family declared that "his spiritual faith was overcome by physical death." Recently Mr. Harold Frederic, the author of that popular novel "The Damnation of Theron Ware," died in England and was treated during his illness by one of the Christian Scientists. We present below what the *Journal* says:

The very recent death of Harold Frederic, the clever novelist and widely-known London correspondent of the *New York Times*, from heart disease, for which he had, until a few days before his death, received "absent treatment" from a certain Mrs. Mills, a Christian Scientist, has attracted very general attention.

It is reported at the inquest, which was adjourned till Wednesday, October 26th, Mr. Frederic's daughter Ruth said her father did not believe in doctors. She added that it was with his consent, but under the influence of others, that Mrs. Mills, a Christian Scientist, was summoned to attend.

Kate Lyon, a member of the Frederic household, testified that the deceased had asked her to call in Mrs. Mills, and the latter came to the house and expounded the system followed by the Christian Scientists. On the following day Mr. Frederic dismissed the doctors who had been in attendance on him.

Most of Mrs. Mills's treatment, continued the witness, was what was known as "absent treatment." At the request of friends of the sick man, the doctors were again summoned, but the patient informed them that when they had previously been attending him he had not followed their directions.

During this same month of October, the death of another gentleman of considerable education and social position, Major Cecil Lester, Instructor at the Sandhurst Royal Military College, occurred in England whilst under the "absent" and present treatment of another female Christian Scientist, a Mrs. Grant; this case has also attracted much attention, and is the subject of an editorial in the *Lancet* of October 15, under the title of unchristian nescience.

From the medical science point of view, the two cases exhibit important differences; from the Christian Science point of view they were alike fit for the practice of absent treatment by persons who knew nothing whatever of the diseases from which the patients suffered, and who evidently considered this a detail of no consequence.

In each case there was an inquest which verified the diagnosis and justified the treatment and prognosis of the medical men originally summoned to the patients.

Frederic was a self-educated, self-made man, with some of the contempt for, or impatience with, the conservative experience of the past which that class of man is apt to entertain. He was of fine physique, and died at the age of forty-two, from cardiac and other complications supervening upon a previous attack of acute rheumatism. The post-mortem examination established the fact that with what medical science would call proper treatment, he would now be alive with an excellent chance for ultimate recovery of good health.

Major Lester suffered from tuberculous peritonitis. His physicians told the family there was little, if any, hope of his recovery, but that much might be done to alleviate his discomforts. Upon this Mrs. Grant was telegraphed to, and she commenced her treatment at once, before seeing the patient, "by what she should call taking up the right thought of the omnipotence and love of God."

In Frederic's case, it was largely the patient himself who insisted upon Christian Science, and secretly neglected other treatment when originally provided. In Major Lester's case it was the friends who, learning the verdict of the physicians, cherished the "rational and reasonable hope" that there might be something in the Christian Science treatment which would lead to recovery. At the inquest on this latter case, the jury added to their verdict riders expressing their entire satisfaction with the treatment and action of the physicians, and "their sense of abhorrence at the so-called treatment of the deceased by Mrs. Grant, as representing the Christian Scientist Society, in not using material means for the alleviation of his suffering."

The contemplation of this sufferer's death-bed leads the *Lancet* "to pass briefly in review the reasons given by the exponent of the Christian Science treatment for the hope that is in Christian treatment—an excellent and taking phrase, but surely a misnomer from beginning to end. Mrs. Grant told the jury that she gave no material remedy to alleviate the pain. Where was the treatment? Mrs. Grant told the jury that she had no experience in the treatment of peritonitis; yet she commenced her treatment directly she got the telegram and before she had seen the patient. Where was the science? Mrs. Grant 'taking up the right thought of the omnipotence and love of God, told the jury that she did not believe that suffering was sent by God, and she contended that Jesus Christ never sent the sick to physicians. If the agony at Calvary for man's redemption was a God-sent reality, and if the Miraculous Healer told the Pharisees that 'they that be whole need not a physician, but they that are sick,' where is the Christianity of the Christian Scientist?

"Though sufficiently vital to demand notice we have no desire here to labor this aspect of the question. We feel it rather to be our duty, our solemn duty, to warn Christian Scientists of the grave responsibility which they incur in regard to the health and lives of their fellow-beings. We must point out that their methods of so-called treatment are ignorant devices, spurious in themselves, and altogether unscientific and opposed to common sense. The Christian Scientist talks as if his (merely human) activities were set in motion and pervaded by an atmosphere of divine potentialities. In an almost flattering way he, as it were, 'stands in' with God, or, as coroner Roumieu bluntly put it at the inquest, he 'tries to usurp the special power which Christ had.' We feel it, further, to be our duty to point out that apart from evils which may result to individual members of the community from it, the Christian Science treatment is neither more nor less than a *fin-de-siècle* fad, a drawing-room cult, and that it is the counterpart of the culpable negligence which has brought the peculiar people of the less educated classes within the range and operation of the criminal law of the country."

---
